# “Smoking paradox” is not true in patients with ischemic stroke: a systematic review and meta-analysis

**DOI:** 10.1007/s00415-019-09596-3

**Published:** 2019-10-29

**Authors:** Bo Li, Dan Li, Jing-Feng Liu, Lin Wang, Bao-Zhu Li, Xiu-Juan Yan, Wei Liu, Kun Wu, Ruo-Lan Xiang

**Affiliations:** 1 Department of Neurology, Beijing Hepingli Hospital, No.18 North Street Hepingli, Dongcheng District, Beijing, 100013 China; 2 Department of Physiology and Pathophysiology, Key Laboratory of Molecular Cardiovascular Sciences, Ministry of Education, and Beijing Key Laboratory of Cardiovascular Receptors Research, Peking University School of Basic Medical Sciences, No.38 Xueyuan Road, Haidian District, Beijing, 100191 China

**Keywords:** Ischemic stroke, Smoking, Poor outcome, Prognosis, Meta-analysis

## Abstract

**Background:**

Ischemic stroke (IS) is a common cause of death from vascular diseases. Studies have found that smoking increases the risk of ischemic stroke, but the association of smoking with the outcome of IS remains unclear. This meta-analysis aims to investigate the effect of smoking on the prognosis of IS.

**Methods:**

We searched four electronic databases including PubMed, EMBASE, Cochrane library and Web of science for papers, published before January 2019. In this meta-analysis, Review Manager 5.3 software was used to calculate for the pooled estimate effect, as well as the inverse-variance method for pooled mean difference (MD) and odds ratio (OR) of incidence in two groups of population.

**Results:**

A total of 14,789 citations were identified during the literature search, 21 studies were included in the meta-analyses after screening. The full-adjusted OR of poor prognostic outcome in smoking and nonsmoking patients with stroke was pooled as 0.96 (95% CI 0.77–1.21), suggested that smoking or not has no impact on prognosis of IS. The pooled MD of onset age between smoking and nonsmoking IS patients was − 10.05 (− 12.91, − 7.19), indicated that smoking causes first onset of IS to occur 10 years earlier.

**Conclusions:**

This meta-analysis showed that smoking was not a protective factor for poor prognosis of IS. Smoking patients with IS are 10 years younger than nonsmoking patients at time of the first onset of stroke.

## Introduction

Ischemic stroke (IS) is the second most common cause of death from vascular diseases and the third major cause of disability worldwide [1]. The previous studies showed that the overall incidence of first-time strokes increased by 11.9% annually (12.4% for men and 9.0% for women) from 1992 to 2015 [2]. In 2015 alone, there were 42.4 million stroke patients nationwide, including 24.9 million IS patients and 3 million deaths from IS. It has been become particularly important to effectively reduce the incidence of IS.

Smoking causes a variety of diseases and kills nearly 6 million people every year (WHO, 2015). Although the prevalence of smoking has decreased in the past 30 years, the absolute number of smokers still increases due to the rapid growth of population, that is, from 721 million in 1980 to 967 million in 2012 [3]. Currently, active smoking is a recognized risk factor for stroke, with 12.4% of accidental stroke patients attributable to current smoking [4]. Studies have found that pre-stroke smoking has a negative or neutral effect on the prognosis of stroke [5–12]. But recent studies reported the so-called “smoker’s paradox”, suggesting that smokers who undergo thrombolytic therapy have better clinical outcomes. This paradox has been found in patients with myocardial infarction and IS treated with intravenous thrombolysis (IVT), and studies have shown that IS patients who experience smoking may have better recovery and better thrombolytic response than non-smokers. Nevertheless, some researchers insisted that the smoking paradox is caused by variances from case to case and that smoking is not an independent prognostic factor in patients with ischemic stroke. The influence of smoking on the prognosis of IS patients, including the influence of passive smoking on IS has not been clearly defined yet.

Based on these conflicting results, the aims of this meta-analysis were to explore the prognostic effects of smoking on IS patients.

## Methods

The authors declare that all supporting data are available within the article (and its online supplementary files). This meta-analysis was performed according to the PRISMA guidelines [13]. We used publicly available published studies, and our study was exempt for approval from Institutional Review Board.

### Eligibility criteria

Eligible trials had to satisfy the following prespecified PICOS criteria: (1) P: ischemic stroke patients; (2) I: smoking; (3) C: no smoking; (4) O: odds ratio of poor prognosis or severity of admission; and (5) S: prospective or retrospective study.

### Search strategy

We conducted a systematic search for articles published before January 2019 without language and data restrictions, through PubMed (Medline), EMBASE (Excerpta Medica Database), Cochrane library and Web of Science computerized databases. The following search terms were used: “ischemic stroke” or “brain ischemia” or “cerebral infarction” or “cerebral vasospasm” or “cerebral angiospasm” or “cerebrovascular obstruction” and “cigarette” or “smoking”. During the search process, we not only used MeSH keywords for retrieval, but also used a broader range of search terms to collect all articles related to this topic. We not only searched the original published articles, but also the references cited in the relevant review articles. In addition, we also retrieved relevant conferences abstracts, reviews and the publications of experts.

### Selection criteria

All the literatures were proceeded to full-text screening by two reviewers. Their detailed examination of the full text would lead the studies to be included or excluded. If there were duplicate studies, the earlier or more detailed publications would be included. If the review contained original published data, it would be also included.

### Data extraction and analysis

One investigator used a pre-designed sheet to extract and document data from eligible studies, including authors, publication year, study design, population, age, male, sample size, grouping and number of people in the group, data including counts and effect estimates, country, follow-up years, title, conclusion. Another investigator independently reviewed to ensure accuracy of data.

### Statistical methods

Review Manager Software version 5.3. from the Cochrane Collaboration (London, United Kingdom) was used to calculate the pooled estimate effect and the inverse-variance method was used to combine effect size. The mean differences (MD) of NIHSS score between the smoking and nonsmoking groups of IS patients were pooled to explore the association of smoking or not with the severity of illness at time of admission to hospital. Meanwhile, the prognosis of IS patients who smoke or do not smoke was studied to produce odds ratio (OR) of poor functional outcome. The between-study heterogeneity was measured by *I*^2^ statistic and Q test. *P* < 0.01 was deemed as significant heterogeneity. We pooled effect size (ES) estimates with significant heterogeneity using random-effects model, otherwise using fixed-effects model.

### Quality assessment and risk of bias across studies

We used the Newcastle–Ottawa Scale (NOS) for assessing the quality of the studies in meta-analyses. We visualized possible publication bias by means of a funnel plot, i.e., ES scatter plots, estimated from single study to compare with their standard deviation.

## Results

### Eligible studies

**Fig. 1:**
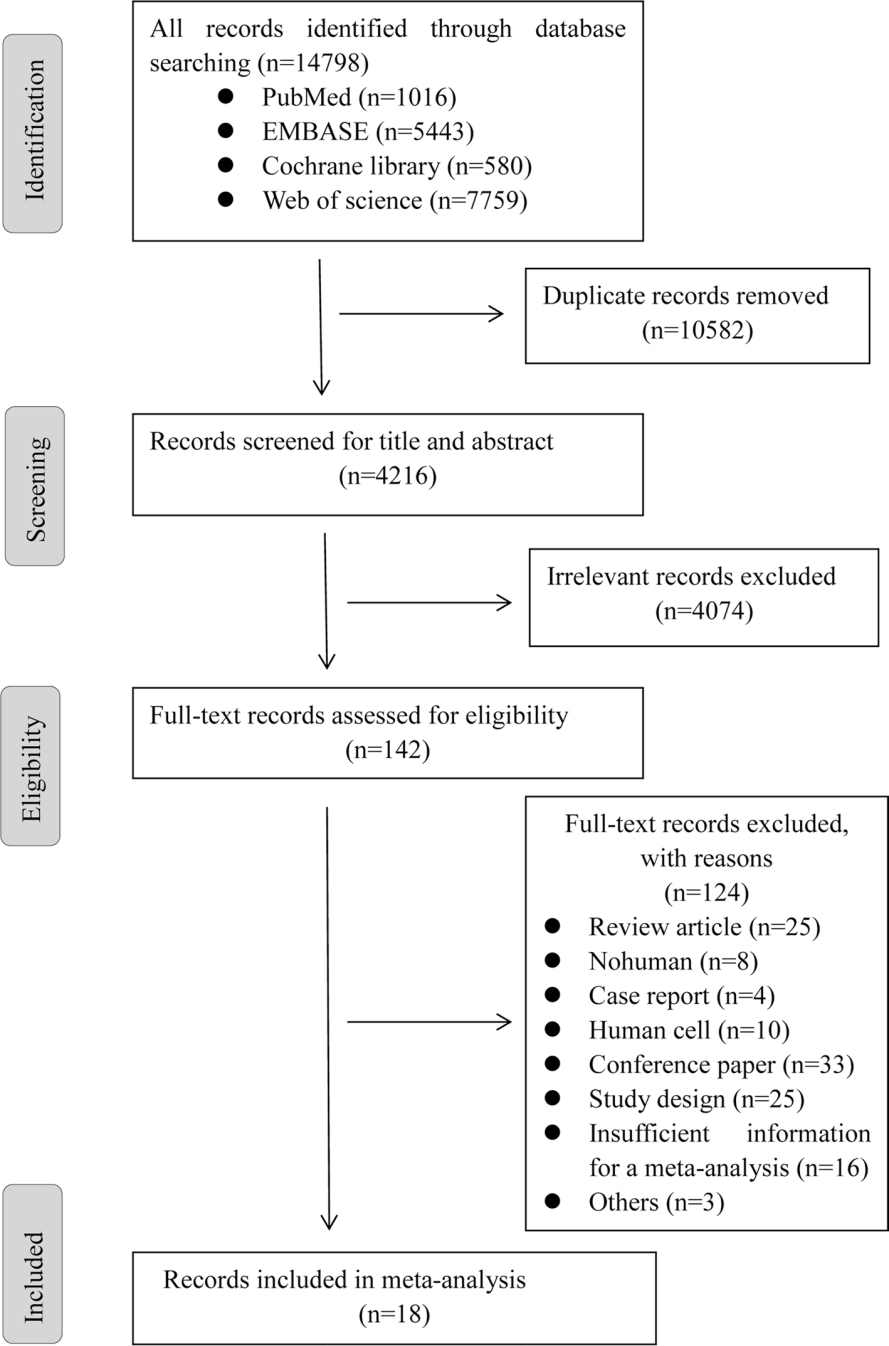
Flowchart of study selection

14,789 related electronic citations were tracked. The flowchart of the study selection is depicted in Fig. 1. As a result, 142 articles were included following title/abstract screening and 124 articles were excluded following full-text artificial selection. Among them were: review article (*n* = 25), non-human (*n* = 8), case report (*n* = 4), human cell (*n* = 10), conference paper (*n* = 33), study design (*n* = 25), insufficient information for a meta-analysis (*n* = 16), others (*n* = 3). In the end, 18 articles were included in the meta-analysis.

### Description of studies

In this meta-analysis, 18 articles with a total sample size of 987,074 were included. The detailed information of each study is listed in Table 1 and the results of evaluating studies quality using NOS scale are listed in Table 2.

**Table 1:** Characteristics of included studies

Study	Years	Study design	Population	Age	Male (%)	Sample size	Country	Follow-up (year)
Ali [14]	2013	Prospective cohort study	Using our hospital’s Get with the Guidelines-Stroke (GWTGStroke) registry, we analyzed patients consecutively admitted with AIS	59.6 ± 13.8	60.0	4305	USA	0.0
Ali [15]	2015	Retrospective cohort study	Using our hospital’s Get with the Guidelines-Stroke (GWTGStroke) registry, we analyzed patients consecutively admitted with AIS	70.3 ± 14.8	48.5	899,295	Canada	0.0
Bejot [16]	2014	Prospective cohort study	All patients diagnosed with a first-ever IS occurring between 1st January 2006 and 31st December 2011 were prospectively identified among residents of the city of Dijon, France (2007 census: 151,543 inhabitants) from the Dijon Stroke Registry	63.3 ± 16.7	65.2	973	France	0.0
Chung [17]	2016	Retrospective cohort study	We retrieved data from TVGHSR on patients who were consecutively admitted and registered between January 1, 2012 and February 28, 2014	74.9 ± 8.9	75.0	60	Taiwan	1.0
Edjoc [9]	2013	Prospective cohort study	The Registry of the Canadian Stroke Network (RCSN) contains data for over 50,000 strokes in Canada. 17 participating sites include all Ontario acute care institutions. A cohort of 20,523 patients was selected for this study from the RCSN	61.8 ± 13.17	52.1	20,523	Canada	1.0
Fekete [10]	2014	Retrospective cohort study	The database of the Mures Uzhgorod Debrecen study was analyzed. Altogether 1049 patients are recorded in the database (603 men)	−	−	716	Hungary	1.0
Glymour [18]	2008	Prospective cohort study	The Health and Retirement Study (HRS) is a national, longitudinal survey of U.S. adults aged ≥ 50 years and their spouses. 10–12 Enrollments occurred in 1992, 1993, 1998, and 2004, staggered by birth cohort	61	−	16,225	USA	9.1
Hou [19]	2017	Case–control study	A dataset from the China Nationwide Retrospective Mortality Survey, conducted from 1989 through 1991, was used. This survey included 1,136,686 all-cause deaths of subjects aged 30 years or older during the years 1986–1988 from 24 urban areas and 79 rural counties randomly chosen from over 2000 counties in China	64.9 ± 10.4	66.7	32,410	China	0.0
Hou [20]	2017a	Prospective cohort study	First-ever ischemic stroke patients hospitalized in the Department of Neurology, West China Hospital, Sichuan University were eligible for this study. A total of 720 first-ever ischemic stroke patients were recruited during 2010–2014	61.5 ± 12.4	100.0	378	China	3.0
Kim [5]	2012	Prospective cohort study	1589 cases of first-ever and recurrent stroke were recruited between 1996 and 1999 from a defined geographical region in North East Melbourne. Both hospital and nonhospital cases were included	−	48.3	1230	Australia	10.0
Kumagai [6]	2013	Prospective cohort study	Patients were enrolled in this study from participants in the Edaravone and Argatroban Stroke Therapy (EAST) for Acute Ischemic Stroke study. The study began in August 2004 and ended in May 2008	71.9 ± 9.7	59.8	660	Japan	0.2
Lee [21]	2015	Retrospective cohort study	Subjects were consecutive patients with first-ever ischemic stroke and without previous functional disability (modified Rankin Scale [mRS] score > 1) who were admitted to Hallym University Medical Center within 7 days of symptom onset between October 2007 and July 2012. The data were populated from the Hallym Stroke Registry, a prospective hospital-based stroke database	65.3 ± 13.5	57.4	1113	Korea	0.2
Ovbiagele [22]	2005	Prospective cohort study	Data from Trials 1 and 2 of the National Institute of Neurological Disorders and Stroke (NINDS) Tissue Plasminogen Activator (tPA) Study were analyzed	64.19	57.0	305	USA	1.0
Ovbiagele [7]	2006	Prospective cohort study	We abstracted data from the IMAGES trial database for this analysis. We identified 2386 subjects with acute ischemic stroke	−	53.5	2386	USA	0.2
Tong [23]	2016	Prospective cohort study	TIMS-China was a national prospective stroke registry of thrombolytic therapy with intravenous alteplase for AIS patients in 67 major stroke centers in China. Between May 2007 and April 2012, 1440 AIS patients treated with IVT were registered in the TIMS-China project	−	60.8	1118	China	0.2
von Martial [24]	2018	Prospective cohort study	This study was based on the Bernese stroke center database, a systematic prospective registry of consecutive patients with ischemic stroke treated at the Stroke Center of University Hospital of Berne, Switzerland. we analyzed all stroke patients who underwent EVT between January 2005 and December 2015. A total of 935 patients were eligible for this study	68 ± 13.9	54.3	935	Switzerland	0.2
Weng [12]	2011	Retrospective cohort study	Patient data were collected from SRICHS. There were a total of 3843 ischemic stroke patients in the SRICHS in 2009. We included 2740 patients	63.9 ± 12.5	62.7	2650	Taiwan	0.0
Zhang [25]	2017	Retrospective cohort study	We retrospectively reviewed the prospectively maintained stroke registry of a single medical center (Xuanwu Hospital) comprising 1910 non-cardiogenic ischemic stroke patients consecutively discharged from January 2013 to October 2014	58.68 ± 11.60	95.4	1792	China	1.0
Total 18 studies				987,074

**Table 2:** The NOS for assessing the quality of studies

Cohort studies			
Reference	Selection	Comparability	Outcome
Ali 2013 [14]	☆☆☆☆	☆☆	☆
Ali 2015 [15]	☆☆☆	☆☆	☆
Bejot 2014 [16]	☆☆☆☆	☆☆	☆
Chung 2016 [17]	☆☆☆	☆☆	☆☆☆
Edjoc 2013 [9]	☆☆☆☆	☆☆	☆☆☆
Fekete 2014 [10]	☆☆☆	☆☆	☆☆☆
Glymour 2008 [18]	☆☆☆☆	☆☆	☆☆☆
Hou 2017a [20]	☆☆☆☆	☆☆	☆☆
Kim 2012 [5]	☆☆☆☆	☆☆	☆☆☆
Kumagai 2013 [6]	☆☆☆☆	☆☆	☆☆
Lee 2015 [21]	☆☆☆	☆☆	☆☆☆
Ovbiagele 2005 [22]	☆☆☆☆	☆☆	☆☆☆
Ovbiagele 2006 [7]	☆☆☆☆	☆☆	☆☆
Tong2016 [23]	☆☆☆☆	☆☆	☆☆☆
von Martial 2018 [24]	☆☆☆☆	☆☆	☆☆☆
Weng 2011 [12]	☆☆☆	☆☆	☆
Zhang 2017 [25]	☆☆☆	☆☆	☆☆☆

Case–control studies			
Reference	Selection	Comparability	Exposure
Hou 2017 [19]	☆☆☆☆	☆☆	☆☆☆

### Meta-synthesis of results

**Fig. 2:**
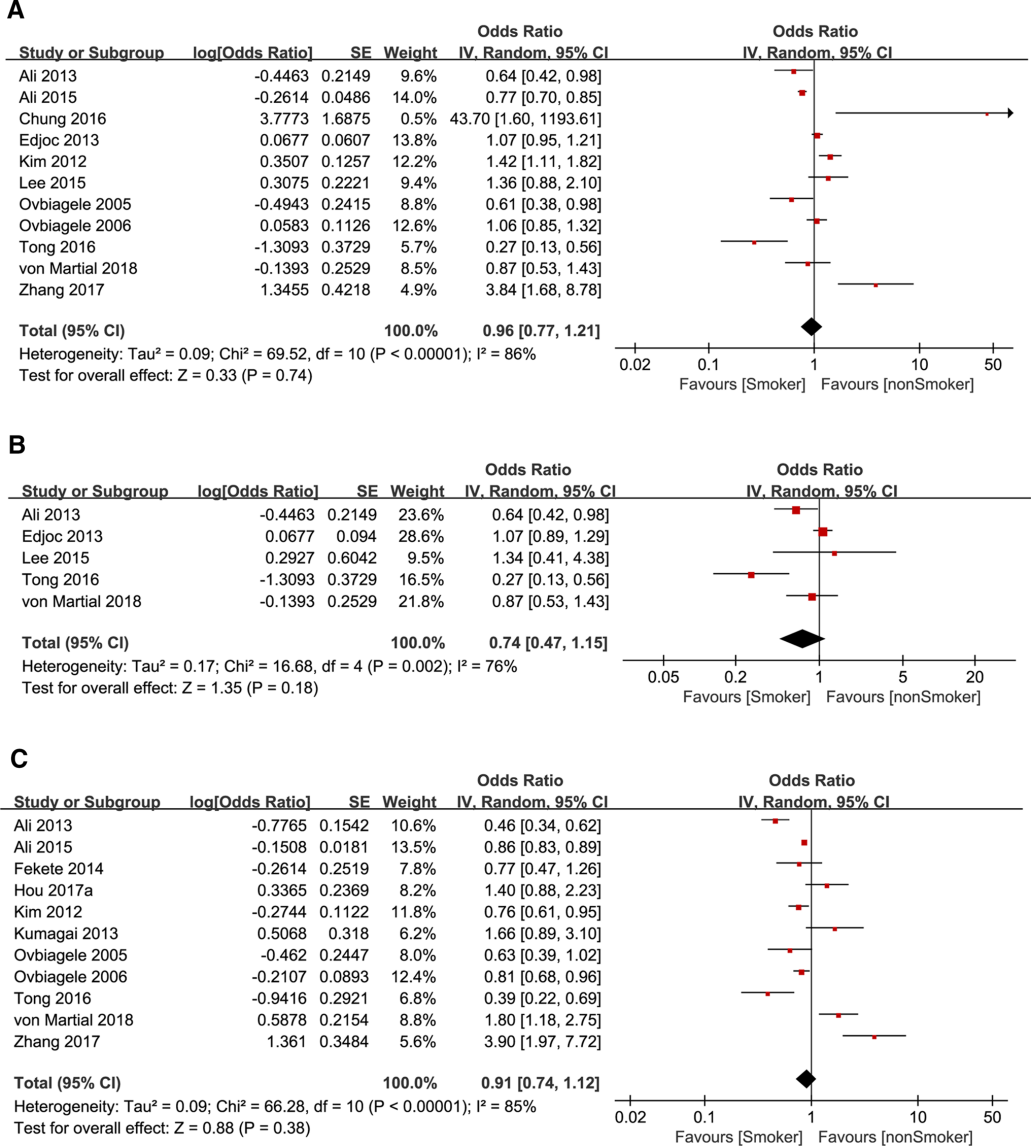
**a** Forest plot of poor prognosis outcome odds ratio (full adjusted) among patients with ischemic stroke, smoking versus nonsmoking. **b** Forest plot of 90-day mortality odds ratio among patients with ischemic stroke, smoking versus nonsmoking. **c** Forest plot of poor prognosis outcome odds ratio (unadjusted) among patients with ischemic stroke, smoking versus nonsmoking

We conducted meta-analysis of 11 studies, and produced a pooled OR (full adjusted) of poor prognostic outcome in smoking and nonsmoking groups of IS patients (OR = 0.96, 95% CI 0.77–1.21, *P* = 0.74 with no statistical significance (SS)). The common adjusted factors included age, sex, BMI and stroke severity at admission. The pooled OR suggested that smoking or not has no impact on prognosis of IS, and smoking is not a protective factor of poor prognostic outcome of IS. *I*^2^ = 86% was deemed as large heterogeneity (Fig. 2a). We carried out a subgroup meta-analyses and pooled OR of 90-day mortality in smoking group with ischemic stroke (OR = 0.74, 95% CI 0.47–1.15, *P* = 0.18 with no SS). *I*^2^ = 76% was deemed as moderately decreased heterogeneity. This overall effect size suggests that smoking or not was not related to the 90-day mortality of IS patients (Fig. 2b). In addition, we also pooled OR of unadjusted prognosis (OR = 0.91, 95% CI 0.74–1.12, *P* = 0.38 heterogeneity *I*^2^ = 85% with no SS). Univariate analysis suggested that even if the covariates such as age, gender, BMI and stroke severity were not adjusted, smoking is not a protective factor for poor prognosis of IS (Fig. 2c). As three of above pooled effect sizes indicated, the smoking paradox is not supported by the fact that smoking or not is not related to poor prognostic outcome of IS.

**Fig. 3:**
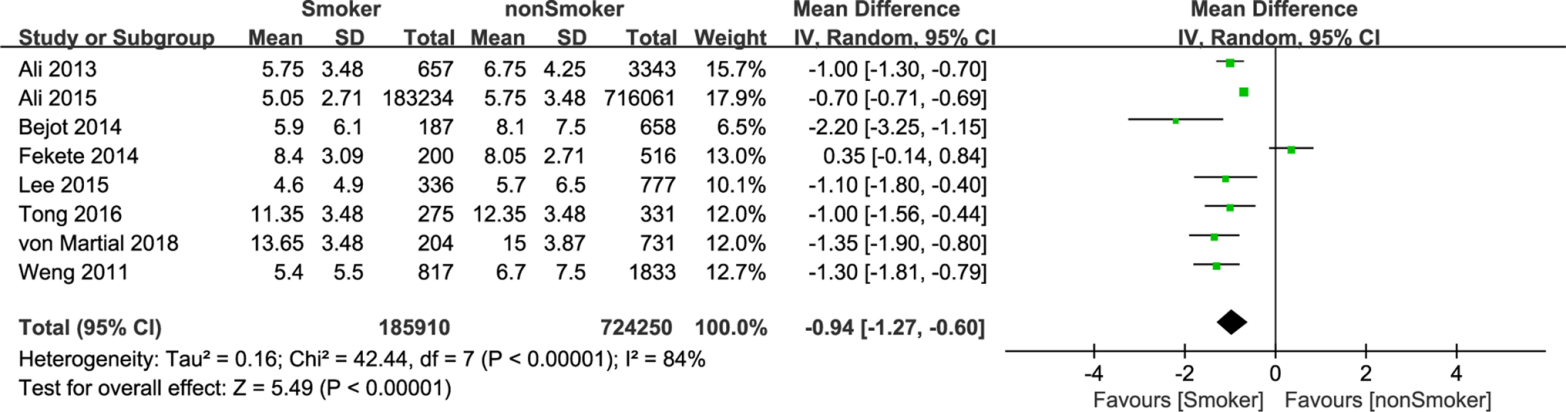
Forest plot of mean difference of NIHSS at admission among patients with ischemic stroke, smoking versus nonsmoking

**Fig. 4:**
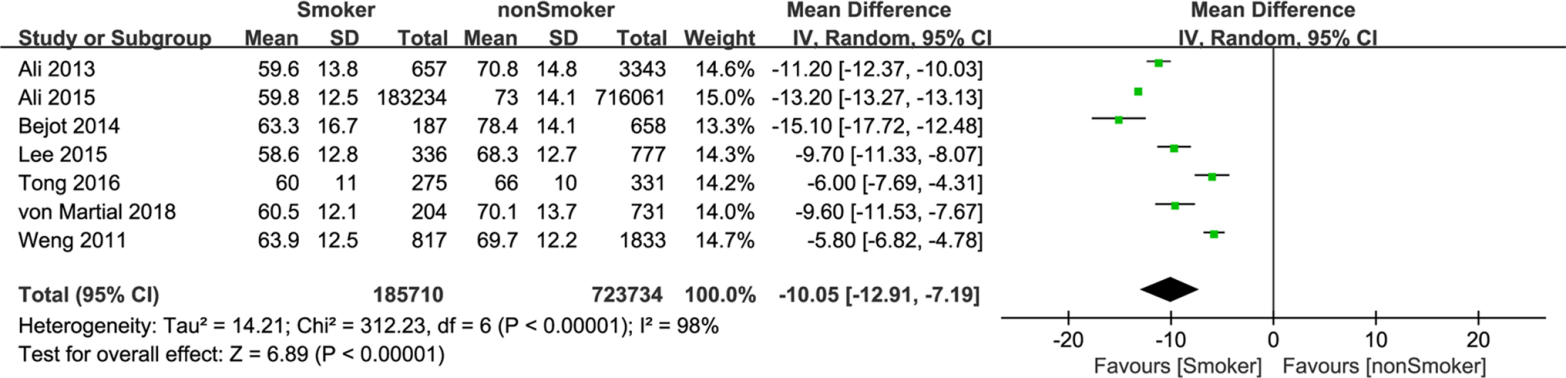
Forest plot of mean difference of age at admission among patients with ischemic stroke, smoking versus nonsmoking

We also studied the state of illness in smoking or nonsmoking groups at the time of admission and combined the MD of NIHSS score between the two groups (MD = − 0.94, 95% CI − 1.27 to − 0.60). It showed the NIHSS score of “smoking group” was smaller than that of the “nonsmoking group” with a standard mean difference of − 0.94, which was statistically significant (*P* < 0.001). Heterogeneity (*I*^2^ = 84%) was large (Fig. 3). Although the effect size of the vast majority of included studies and the pooled overall effect size supported the notion that patients with cigarette smoking were less severe than patients without cigarette smoking at the time of admission, we believed this does not mean smoking is beneficial to the condition of ischemic stroke, as the NIHSS score is associated with a variety of other factors. We made comparison of onset age between smoking and nonsmoking IS patients (except Fekete 2014 for the absence of age data). The pooled MD was − 10.05 (− 12.91, − 7.19), indicated that smoking brings forward the age of first onset of IS by 10 years. You can see from Fig. 4.

### Publication bias and study quality

**Fig. 5:**
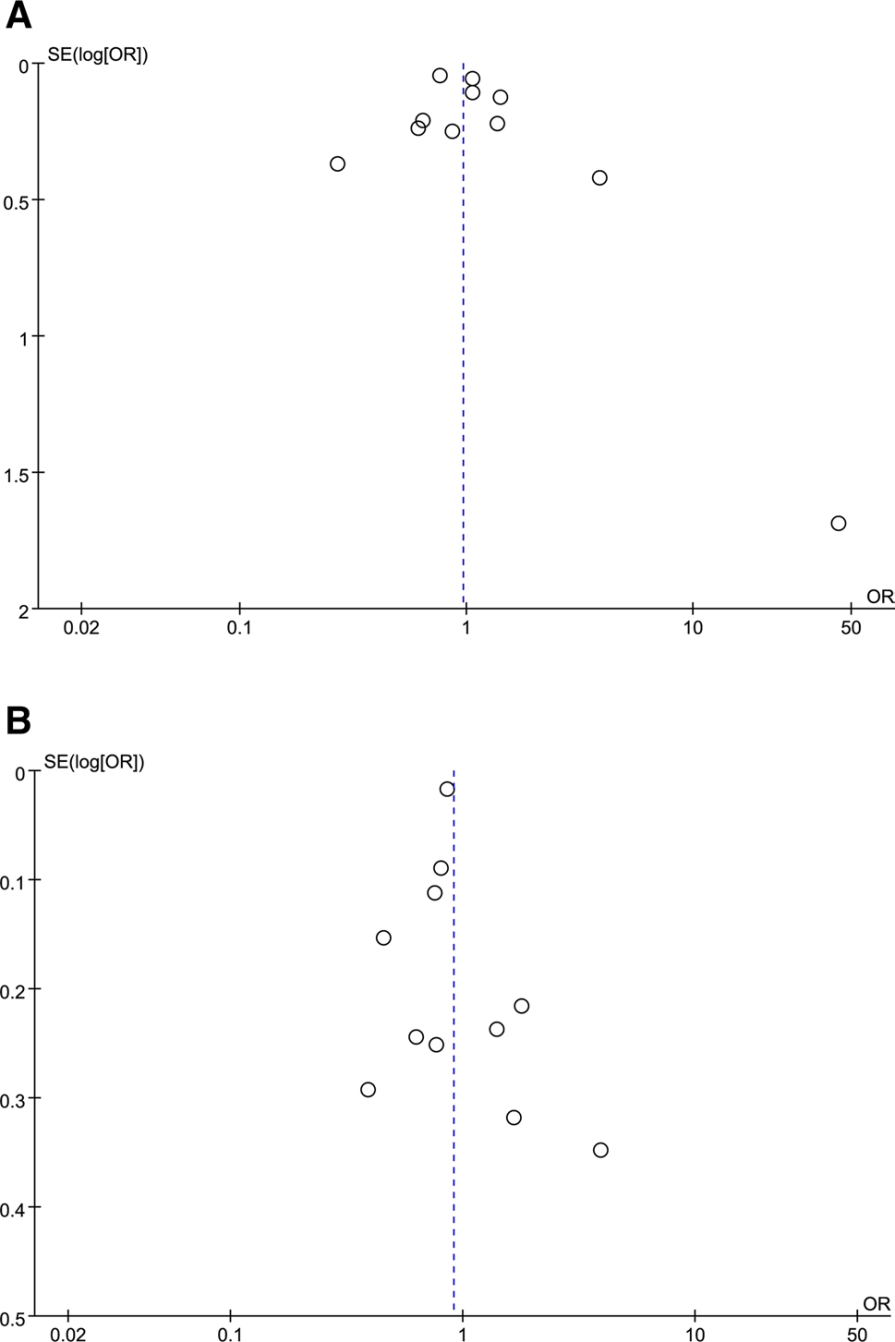
**a** Funnel plot of poor prognosis outcome odds ratio (full adjusted) among patients with ischemic stroke, smoking versus nonsmoking. **b** Funnel plot of poor prognosis outcome odds ratio (unadjusted) among patients with ischemic stroke, smoking versus nonsmoking

The evaluation of quality was carried out for all 18 included studies and the results of NOS scale are shown in Table 2. To explore publication bias, we drew the funnel plot for the effect size of all the studies in Fig. 2a. The funnel plot visually was relatively symmetrical, as shown in Fig. 5a. The pooled effect size of the rest 10 studies varied from 0.96 to 0.95, with no SS, and there is not any change in statistic *I*^2^, even after that the study “Chung2016” was excluded due to deviation of its effect size too far. Therefore, we believed Chung2016 was insensitive to this comparison.

We also drew the funnel plot for the effect size of all the studies in Fig. 2c. The funnel plot visually was relatively symmetrical with little bias, as shown in Fig. 5b.

As regards to publication bias of other comparisons, the funnel plot was of limited use due to the small number of studies evaluated.

## Discussion

Eighteen studies comprising 987,074 patients were included in this meta-analysis. The results indicate that smoking is not a protective factor for poor prognosis in patients with IS, and the “smoking paradox” is not true. At the same time, it is found that smoking IS patients are 10 years younger than those who do not smoke, which further proves that smoking is an important risk factor for IS. Many studies are actively exploring the relationship between smoking and IS, and this possible link will have important implications for clinical practice and public health.

Smoking has long been considered as an independent risk factor for IS. Many studies reported smoking is an independent risk factor for poor prognosis in patients with ischemic stroke. Compared with patients who never smoked, those who smoked during or before stroke had a greater risk of death or recurrent vascular events [5]. Nevertheless, the contradictory results, namely smoking paradox, were reported in studies by many researchers who investigated the relationship between smoking and prognosis of stroke patients. Some of them reported that smoking in stroke patients may be independently associated with excellent clinical outcome after Endovascular Treatment (EVT) [24]. Tong et al. [23] recently reported that noncardioembolic stroke may be independently related to good outcome in smoking patients treated with Intravenous thrombolytic (IVT). The researchers believe that smokers may have better ischemic preconditioning due to elevated levels of carbon monoxide in the plasma and intermittent hypoxia [26]. Smokers may be supposed to have a better cerebral collateral supply as a further explanation for paradoxical association with clinical outcome, but collateral supply did not differ between groups in our study. These contradictory results prompted us to assess the prognostic impact of smoking and nonsmoking on patients with ischemic stroke to determine the correlation between smoking and IS prognosis. After adjusting factors such as age, sex, BMI and severity of stroke at admission, we found that smoking was not associated with the prognosis of IS. Even if covariates such as age, sex, BMI and severity of stroke were not adjusted, our results also exhibited that there was not correlation between smoking and the prognosis of IS. This suggested that the paradox “smoking is beneficial to the prognosis of ischemic stroke” is not valid. Smoking is not a protective factor for the prognosis of ischemic stroke. Those contradictory results might derive from the differences of study size.

This meta-analysis also indicated that smokers were 10 years younger than non-smokers at the onset of stroke, suggesting that smoking may cause the first onset of stroke to occur significantly ahead of time. A large number of studies have found that smoking produces more than 4000 gases, including carbon monoxide and nicotine [27]. Carbon monoxide can replace oxygen in hemoglobin, thus reducing the release of oxygen and directly reducing the oxygen supply to tissues and organs [28]. In addition, those toxic chemicals in cigarette smoke, such as nicotine, can lead to vascular endothelial dysfunction and inflammation, which ultimately leads to the development and acceleration of the atherosclerosis process. Smoking patients are assumed to have an increased hematocrit, platelet activation and aggregation, vasoconstriction and circulating fibrinogen [29–31]. Thus, smokers may have more thrombogenic than atherogenic vessel occlusion. The location of vessel occlusion may also play an important role. Smoking patients are more likely to have aortic occlusion, which directly affects the treatment effect and prognosis. In addition, smoking increases oxidative stress with the loss of the protective effect of NO tips the cellular balance towards a proatherogenic and prothrombotic milieu. All of these increase the risk of IS. Therefore, smoking is a risk factor for IS, making the patient suffering from IS 10 years earlier. This also explains why this meta-analysis reveals that the NIHSS score of smokers at admission is lower than that of non-smokers, and pooled MD = − 0.89, and most of the six included studies indicate that smokers are less ill than non-smokers. In five studies, NIHSS scores of smoking patients with ischemic stroke were lower than those of nonsmoking patients [10]. Only one study found that smoking patients with ischemic stroke had higher NIHSS scores than nonsmoking patients. Smoking patients with IS seem to be less ill. By the way, the NIHSS scores reported by these studies at admission are unadjusted for any of the influential variables. The NIHSS scores are related to many other factors, such as age. Significant differences in age between smoking patients and nonsmoking patients may be an important factor contributing to differences in NIHSS scores. The younger patients had lower NIHSS scores because their basic physical condition is relatively better. Smoking patients are 10 years younger and are less likely to have other traditional vascular risk factors compared with non-smokers. Although the study found that smokers’ NIHSS scores were better than non-smokers, these age differences may partly explain the beneficial findings for smokers, as older age is often associated with worse physical conditions [24, 32]. Considering differences in baseline characteristics are essential for discussion of “smoking paradox” in stroke patients, the age is one of the important factors. According to a comprehensive consideration of factors, such as age, this meta-analysis determined that not only smoking was not related to the prognosis of IS, but also cause the first onset of IS to occur 10 years earlier. We believe that the smoking paradox is not true. We strongly recommend giving up smoking. The government should increase the nationwide education of tobacco harmness to health and increase the investment and intensity of tobacco control.

### Strengths and limitations of study

The strength of this paper is that we synthesized 18 studies, comprising nearly 1 million samples, many of which are of large scale. Therefore, the evidence provided by us in this meta-analysis is sufficient to give a reliable estimate of the relative risks associated with smoking. Our analysis also has some limitations. First, between-studies heterogeneity can be increased due to definition of smoking that varies from study to study included. Second, between-studies heterogeneity can be increased due to paucity of data in subgroup analysis of stroke patients who quit smoking or continue to smoke after admission and during follow-up.

## Conclusions

The “smoking paradox” is not true. Smoking has no protective effect on the poor prognosis of IS patients. Smoking IS patients are 10 years younger than nonsmoking IS patients at the time of first onset of stroke. Smoking is one of the most preventable causes of IS risk. This requires the government to strengthen tobacco control and raise people’s awareness of reducing and quitting smoking.

## Abbreviations

ES: Effect size
IS: Ischemic stroke
MD: Mean differences
NIHSS: National Institute of Health Stroke Scale
OR: Odds ratio
SS: Statistical significance
